# Developing Healthcare Leaders: Perspectives on Critical Competencies, Current Gaps, and Training Recommendations

**DOI:** 10.7759/cureus.108705

**Published:** 2026-05-12

**Authors:** Sonum Tharwani, Madeleine Laughon, Dalia Namak, Christina Shenvi

**Affiliations:** 1 Internal Medicine, Duke University Health System, Durham, USA; 2 Medical Education, University of North Carolina at Chapel Hill, Chapel Hill, USA; 3 Healthcare, Humana, Chapel Hill, USA; 4 Emergency Medicine, University of North Carolina at Chapel Hill, Chapel Hill, USA

**Keywords:** healthcare leadership, leadership development, medical education, physician leadership, system leadership

## Abstract

Introduction

This study sought to define the leadership skills and competencies that are essential to physician leaders and current gaps in physician leadership training. The study also aimed to identify the skills that current physician leaders would have liked to develop earlier in their careers and generate recommendations for successfully integrating leader development into medical education.

Methods

This qualitative study explored healthcare leader development through semi-structured interviews with 14 healthcare executives from major healthcare systems in the Southeastern United States. Participants were selected based on their high-level leadership roles. Interviews were conducted virtually and focused on three areas: critical leadership skills, reflections on early career development needs, and strategies for integrating leadership training into medical education. Transcripts were thematically analyzed by three independent investigators to identify recurring themes and insights.

Results

Healthcare leaders identified six essential leadership skills: adaptability, empathy and gratitude, relational leadership, change leadership, project management, and knowledge of the healthcare system. Common gaps that emerging leaders should address include relational leadership, redefining leadership beyond hierarchy, self-awareness, understanding the healthcare system, and financial acumen. To prepare future physician leaders, interviewees emphasized the need for training in professional identity formation as a leader, experiential learning, interdisciplinary collaboration, self-awareness, and resilience. Several themes, including relational leadership, healthcare system knowledge, self-awareness, and business acumen, emerged across multiple domains.

Conclusions

Healthcare leadership requires more than clinical expertise alone. The role demands a blend of interpersonal, adaptive, and technical skills to navigate complex challenges. Given the growing demands placed upon healthcare leaders, medical institutions must integrate structured leadership training early in physicians’ careers. Preparing future leaders through intentional education and mentorship will help ensure that the healthcare system remains effective and responsive amid significant external pressures and internal transformations.

## Introduction

While clinical expertise saves lives and directly impacts individual patients, leadership is what transforms healthcare systems, drives innovation, and supports the long-term success of organizations and care delivery systems. To succeed, healthcare organizations require leaders with advanced skills and capabilities. As organizations navigate complex regulatory environments, technological advancements, and financial pressures, effective healthcare leadership demands skills that extend far beyond traditional clinical expertise [1]. The coronavirus disease 2019 (COVID-19) pandemic further highlighted the need for healthcare leaders to balance technical competencies with emotional intelligence and the ability to foster collaborative environments while leading with empathy [2].

While some healthcare professionals pursue additional degrees, such as master’s degrees in business administration or healthcare administration, to develop their leadership skills [3], this path is not feasible for all. Additionally, while most health systems and academic medical centers offer professional development programming, these programs have been criticized for lacking a theoretical foundation and for focusing too narrowly on cognitive domains while overlooking other crucial leadership elements [4]. It also remains unclear which components of these programs effectively contribute to leader development [4]. Despite the availability of well-established healthcare leadership frameworks, there is a lack of evidence that captures the wisdom, expertise, and experiences of practicing executives to inform leadership training design, particularly in the post-COVID era, when leadership demands have expanded beyond traditional competencies.

This qualitative study addresses the aforementioned gaps by exploring the perspectives of healthcare leaders within a large academic, multi-hospital system that includes both urban and rural hospitals and serves diverse patient populations. We investigated both essential leadership competencies and critical knowledge areas that leaders wish they had acquired earlier in their careers. Furthermore, by drawing insights from leaders with varying educational backgrounds (MD, MD/MBA, MBA), we aimed to gather their perspectives on how to better integrate leadership development into medical education. Our focus is on leader development and the growth of individuals into leaders, as we explore personal competencies, experiences, and the developmental journey of healthcare leaders, rather than the more general process of enhancing leadership skills.

## Materials and methods

Study design and ethics statement

This interview-based qualitative study thematically categorized the essential leadership skills in healthcare, identified competencies that could be acquired earlier, and explored strategies for integrating leadership training into medical education. The study was reviewed and deemed exempt by the University of North Carolina (UNC) Institutional Review Board (#23-3243). All procedures were conducted in accordance with the ethical standards of the institutional and/or national research committee and with the 1964 Declaration of Helsinki and its later amendments, or comparable ethical standards. Informed consent was secured from all participants, including consent for video recording, which facilitated transcription and thematic analysis. The research was conducted over six months, divided into two three-month phases: data collection and analysis. During the data collection phase, semi-structured interviews were conducted with healthcare executives across the state. To allow participation among geographically dispersed participants, interviews were conducted virtually via video conferencing. No funding was obtained for this research.

Participant recruitment and interviews

Thirty-one healthcare executives from prominent North Carolina healthcare systems, including Wake Forest, the University of North Carolina, Duke University, Atrium Health, and Novant Health, were identified as potential participants based on their leadership roles and influence within their respective institutions. Participants were selected using purposive sampling based on their institutional role and domain expertise relevant to the research questions. Senior executives were identified and recruited by the research team based on their leadership responsibilities and direct involvement in the areas under investigation. Efforts were made to ensure a diverse representation of hospital leaders, including chief officers, department chairs, associate deans, hospital presidents, and non-physician administrative leaders.

Of the 29 leaders successfully contacted, 15 were unable to participate: seven did not respond, and eight either declined or were unable to schedule an interview. The final study cohort consisted of 14 participants. Semi-structured interviews, each approximately 20 minutes in duration, were conducted virtually by members of the research team with the leader. Both video and audio recordings were used for accurate transcription. Interviews were structured with three core topics to explore healthcare leadership dynamics: (1) identification of critical leadership skills in healthcare, (2) reflection on the leadership skills that participants wished they had developed earlier in their careers and key lessons they had learned through their leadership roles, and (3) strategies for medical students and emerging leaders to cultivate leadership skills during training.

Data processing and analysis

Interview transcripts were manually reviewed and verified for accuracy by the interviewers before being independently coded by three members of the research team. We performed the thematic analysis using the software Dedoose (Dedoose, Hermosa Beach, CA). Dedoose’s analytic features allowed us to systematically identify primary themes, with subthemes emerging based on frequency and consistency across interviews. Discrepancies in coding were resolved through iterative discussions until consensus was reached, enhancing the dependability of the findings. This approach enabled a rigorous and structured synthesis of the interview data.

## Results

Demographics

The study included 14 healthcare executives (nine women, five men) from across North Carolina. Participants represented diverse educational backgrounds: nine held MDs only, three held MD/MBA dual degrees, one held MD/MPH, another MD/PhD/MBA credentials, and two participants held business degrees, including an MBA and MSN/MBA. Thematic analysis revealed the top themes in the areas of essential leadership skills, leader skill development gaps, and opportunities to develop physician leaders. The themes are presented in Table 1 and are discussed below with representative quotes from the participants.

**Table 1:** Summary of key themes that emerged from the thematic analysis of the structured interviews

Structured interview topic	Key themes
Essential skills required by leaders in healthcare	Adaptability and flexibility
	Empathy and gratitude
	Relational leadership
	Change leadership
	Project management skills
	Knowledge of the healthcare system
Common skill gaps among emerging leaders and in their own past	Collaborative leadership beyond hierarchy
	Self-awareness and self-management
	Systems-based thinking skills
	Financial acumen
	Relational leadership and managing people
Training opportunities to effectively develop future leaders	Professional identity formation as a leader
	Experiential learning
	Interdisciplinary collaboration
	Structured development of self-awareness
	Resilience

Essential leadership skills

Participants identified six key themes that emerged consistently across interviews as essential leadership skills: (1) adaptability and flexibility, (2) empathy and gratitude, (3) relational leadership, (4) change leadership, (5) project management, and (6) understanding of the healthcare landscape and system. Item six was also identified in the list of leader skill gaps and is discussed in that section. Here we expand on the themes discovered here and provide exemplary quotes from participants.

1. Adaptability and Flexibility

In the wake of the COVID-19 pandemic and the more recent rapid emergence of artificial intelligence and telehealth, over half of the interviewed healthcare leaders described the current landscape as increasingly complex, emphasizing adaptability and flexibility as essential leadership qualities. Quotes from participants included:

"Healthcare is ever-changing and ever-evolving. We need to be able to evolve with it and not always cling to the past. Right now, the hot topic is artificial intelligence. How are we going to use it? How are we going to train our learners to use it effectively and understand its limitations as well as its possibilities?"

"...Flex with the system, flex with the changes that come. Probably one of the best examples of that in healthcare right now is how we had to drastically change how we're able to deliver care in the face of natural disasters, global pandemics, and surges in illness."

Leaders who demonstrated agility were deemed better equipped to guide their teams through significant change and uncertainty.

2. Empathy and Gratitude

Empathy and gratitude are not traditionally listed among key leadership competencies or traits. However, many participants in our study linked empathy to improved team morale and reduced burnout, suggesting that leaders who demonstrate genuine concern for their teams' emotional well-being help foster a more supportive work environment. Quotes from participants with context provided are provided here:

It is important to "see things through others' eyes," and "everyone has very different experiences,"

"More than ever, empathy is important."

Gratitude also stood out as a key leadership trait.

"Acknowledging how exhausted our medical staff and teammates are" and expressing "gratitude wherever they can find opportunity to do so.”

"It takes a village to do what you're doing. Gratitude is super important."

3. Relational Leadership

Relational leadership is a people-centric approach that focuses on the need to foster connections, collaboration, and trust. Within this framework, teamwork, communication, and emotional intelligence were highlighted as essential components. Many participants stressed that leadership in healthcare must be a collective effort.

"Nothing happens within a vacuum, and the sole leader concept is not supported."

“See[ing] other people’s perspectives and understand[ing] the goals and constraints of other people’s work while also learn[ing] from them what it is will make the whole system more successful.”

"The people who I absolutely admire, and the people who I aspire to be as a leader myself, are the people who don't lead for the power, for the prestige, for any of those reasons...they lead because they care so deeply about the people."

While teamwork requires inclusive decision-making and recognition of individual roles, communication skills were repeatedly described as the foundation of productive relationships. Leaders also stressed the value of walking around and checking in on others while they are working to build rapport, understanding their roles, and addressing real-time concerns.

“Too often decisions are made without truly understanding what happens in the clinic, on the inpatient service, in the OR.”

"I work in a system where we have 86 different, unique programs... what seems like it should totally work for a radiologist or internal medicine doctor... doesn't make any sense at all for an Ob-Gyn or Surgeon."

4. Change Leadership skills

Change leadership encompasses the need to identify necessary changes, set a vision, gain buy-in, create a concrete plan, and deliver results. The urgency for innovation was underscored by the strain on healthcare systems, particularly after COVID-19. Leaders emphasized boldness in addressing challenges, cautioning against learned helplessness, and advocating for an environment of possibility rather than passivity by “not just settling to the lowest common denominator.”

“We need leaders who...will fight to solve the big problems even if they are problems that are being faced in many places." 

"You need to be able to cast a compelling vision of the future and provide the pathway to achieve that future."

“This is the time in healthcare like no other…We have to innovate, and everybody's tired from COVID...and the inefficiencies in healthcare, and nobody's getting paid for the work that we do, so now is that time where our leaders have to both be able to believe and see how things ought to be, and then seize this moment…to make it better for our patients, for our providers and make it more, sustainable from a cost perspective long term. So, because of that, change leadership is huge.” 

Communicating a clear vision and executing it with an innovative mindset emerged as a cornerstone of effective change leadership, yet participants agreed that vision alone is insufficient. One respondent stressed the importance of following through and delivering, warning that hollow promises quickly erode trust. In large, complex organizations, successful execution requires breaking down ambitious goals into concrete steps and incremental wins to secure buy-in and demonstrate real progress.

Authenticity and the ability to inspire others also arose as essential leadership qualities for change management, particularly as healthcare staff face hopelessness and burnout, making it critical for leaders to motivate people and give them hope. They emphasized that trust, not charisma, drives the ability to inspire others.

“The real trick is getting someone to do something you want done because they want to do it. An effective leader gets you to do the things that need to get done because you're motivated to do it yourself. That's a real skill."

"If people buy into you, then they'll buy into your ideas."

5. Project Management

Strong project management skills were seen as essential for productivity, particularly in ensuring initiatives reach completion. Effective project management relies not only on well-structured teams but also on purposeful meetings. Establishing clear objectives, maintaining focus during discussions, and tracking progress were all viewed as necessary to achieving meaningful results. Formal business training, such as an MBA, was considered valuable for refining project management skills. Beyond offering technical skills, this type of education reinforced the importance of defining success, setting measurable goals, and maintaining accountability throughout a project's lifecycle.

"I didn’t have many role models... a lot of dysfunctional meetings and dysfunctional teams."

"Doing the MBA was very helpful… to be outcome-oriented and know what success looks like." 

Leader skill gaps

Five key themes emerged after leaders reflected on skill gaps present today in junior faculty and on skills they wished they had learned earlier in their careers: (1) redefining leadership, (2) self-awareness and personal development, (3) system-based thinking skills, (4) financial acumen, and (5) building relationships and managing people. Item 5 was also identified as an essential leadership skill and was discussed in the section above. 

1. Collaborative Leadership Beyond Hierarchies

Healthcare leaders' reflections revealed an evolving understanding of leadership that challenges traditional hierarchical models in medicine. Leaders consistently emphasized the importance of leading from behind.

"Not…trying to lead from the front but elevating other people to be in the front."

“Leadership is so much more diplomatic or democratic than authoritarian. Medicine historically has probably been more of an authoritarian, hierarchical training system..… Strong leaders that I emulate are those who are diplomatic and seek feedback. They find people who are experts in their field who help them, lift all thoughts as opposed to trying to make all decisions for everybody."

"I have to learn how to identify talent, magnify their talent, get out of the way, set the vision, help people see it, lift them up, make it easy for them to be successful, and then they are incredible."

"The ability to convey authentically, build trust, and show your human side even when disagreeing - is, in fact, a critical skill…[and is] so important for effective leadership."

Active listening emerged as a key skill. One experienced leader shared that they speak less in meetings currently than earlier in their careers and try to allow others to speak up first to avoid swaying the conversation.

2. Self-Awareness and Self-Management

These were described as foundational leadership skills that leaders wished they had developed earlier. Leaders first emphasized that effective leadership begins with self-management, particularly in challenging situations. Key aspects included understanding one’s own mind and managing one’s thoughts and emotions. One leader emphasized the value of formal self-assessment tools to understand and build on their strengths while working to minimize or mitigate their personal weaknesses. Others discussed building a team with people who have complementary skills. Leaders also emphasized understanding personal motivations:

"It is not about the title, it's about what drives you."

It is important to "not act out when you're stressed or mad."

3. Systems-Based Thinking Skills

Participants emphasized the importance of seeing beyond individual roles to comprehend organizational complexity. Seasoned leaders consistently observed that junior faculty struggle to develop a broader organizational perspective.

"The bulk of the problems come not from individuals, though that's who we tend to blame, but from systems. Too many systems have been set up to fail. As leaders, how do we fundamentally think about [the] ability to understand the system and then understand our role in removing obstacles?"

"Identify what your goals are and how you can put that into action [to] benefit the most people and the objectives of your system. I don't think that I necessarily saw that element of leadership of the system piece early in my career."

"It's not just about you and your location in your department and your division. Take a step back and see how this impacts the other folks around you."

Leaders must adopt a systems-level perspective that considers how different components of healthcare interact. They stressed the need to move beyond siloed thinking, advocating for coordination across departments, alignment of resources, and improvements in both patient outcomes and system sustainability. Leaders must also stay informed about external factors shaping the industry, such as policy changes, political shifts, technological advancements, shifting patient demographics, and evolving care and reimbursement models.

4. Financial Acumen

Participants emphasized both the fundamental importance and the traditional hesitancy of physicians to engage with the financial aspects of healthcare. One leader highlighted the inherent tension between clinical ideals and financial realities:

"The cynical thing is that finance drives everything, and your idealistic visions or goals are tempered by finance.”

Participants emphasized that physicians' traditional disengagement from financial matters has created a problematic vacuum. One leader highlighted how physicians should be comfortable with finance to engage in decision-making informed by patients’ bedside experience, emphasizing that physicians cannot focus on patient care in isolation. Advice that one leader received from a mentor when she decided to pursue an MBA was to focus on finance so that she could "know it well enough to know the right questions." These perspectives emphasized three key aspects: the inherent tension between financial constraints and clinical priorities, the current gap in physician financial literacy that limits their decision-making influence, and the value of combining formal financial education with practical experience. Leaders consistently expressed that early development of financial understanding is about maintaining the physician's voice in decisions that impact patient care delivery. 

Opportunities for leader development during medical education

One leader shared a common misconception among physicians, "that just because they're smart and talented, leadership should come naturally. They don't understand why they aren't just in charge or why no one is listening to them sometimes." This demonstrates the need for intentional leader training to bridge skill gaps. Five critical themes emerged regarding the integration of leadership education into medical training: (1) inherent leadership responsibility and professional identity, (2) experiential learning, (3) interdisciplinary collaboration, (4) self-awareness development, and (5) building resilience.

1. Inherent Leadership Responsibility and Professional Identity as a Leader

A foundational insight was that leader education must begin with helping medical students understand their role as leaders, regardless of practice setting or career path. One interviewee highlighted the critical need to actively cultivate the professional identity of leadership early in medical education:

"If nothing else, it is making sure that our students realize that whether you want to be a leader or not, the world will see you as a leader. Your community will look to you as a leader... especially if you are in a small town, the community needs you, and so [students] need to develop those skills."

2. Experiential Learning

The interviews strongly supported experiential learning as the primary vehicle for leader development. Participants advocated for practical leadership opportunities through activities such as managing student organizations, serving on committees, contributing to research teams, or organizing community health initiatives. One participant notably observed that skills developed in smaller settings translate to larger roles:

"The skills you need to be an effective leader at [your school's free clinic] are the same skills you need to be a leader anywhere. You will just have a bigger platform and bigger budget in future roles."

"The most growth comes from real-world experience, so seeking out mentors, taking on projects, and being reflective about your own strengths and weaknesses is the best way to grow, but medical schools could definitely do more to nurture this growth in students."

3. Interdisciplinary Collaboration

Structured interdisciplinary or interprofessional learning experiences emerged as a critical training opportunity. Participants described successful models of interdisciplinary education, including programs where interprofessional teams collaborated on complex patient cases. As one leader observed,

"We really need more interprofessional education events where we're really getting in everyone's mindset of what it takes to be part of a physical therapy program, what does it take to be a social worker? I think it's hard to be a healthcare leader without understanding all the parts."

4. Development of Self-Awareness

Self-awareness emerged as a critical but often overlooked aspect of leader training. Personal style recognition was particularly noted as foundational to leadership effectiveness. Leaders consistently emphasized the need for structured approaches to understanding individual leadership tendencies. Responsibility awareness was the second component. Participants described how effective leadership requires understanding one's role in organizational problem-solving beyond immediate clinical duties. This was captured by one leader's perspective: "I may not have caused this issue, but it is my responsibility to fix it.” This perspective emphasized how self-awareness enables leaders to better understand both their responsibilities and others' experiences.

5. Building Resilience

Healthcare leaders emphasized resilience as a vital component of leader development, with particular concern about its decline among recent graduates, where one educator observed that their ability to cope with hard work has been difficult. They saw resilience not just as personal coping but as a crucial leadership skill that enables adaptability and effective problem-solving under pressure.

In terms of implementation, one interviewee offered a compelling example of structured, comprehensive leadership training piloted for anesthesia interns through a dedicated, intensive weekend retreat. While this program proved successful with interns, the educator strongly advocated for earlier implementation, stating,

"Until such comprehensive programs become standard in medical education, the responsibility often falls to students. Medical students who don't have exposure need to seek it out themselves. If you read 10 books in a year, one or two ought to be on leadership skill development."

This final insight underscores both the critical importance of systematic leadership education in medical schools and the current gap that students must actively bridge to develop these essential skills.

## Discussion

Clinical expertise provides the foundation for healthcare delivery, yet leadership capabilities enable system-wide transformation and innovation. This analysis of 14 healthcare executives’ experience, perspectives, and insights reveals a fundamental truth: successful healthcare leadership requires interpersonal, adaptive, and technical competencies that traditional medical education has not systematically addressed. As healthcare organizations confront challenges such as workforce shortages, technological disruption, and financial pressures, conventional leadership development approaches have proven insufficient [2].

Our findings identify five essential leadership competencies that represent a clear shift away from conventional frameworks that emphasize technical and operational skills. Most notably, empathy and gratitude emerged as distinct, essential traits that participants directly linked to operational success. While previous research has documented the importance of emotional intelligence in healthcare leadership [1,5], our participants provided concrete examples of how empathy influences team performance and organizational outcomes.

From hierarchy to collaboration

Healthcare leadership has undergone a fundamental transformation from hierarchical authority to collaborative influence. Participants consistently described the importance of “leading from behind” and elevating others to take the lead, representing a significant departure from medicine’s traditionally top-down culture. This transformation requires competencies that medical training currently does not adequately provide.

The emergence of systems thinking, defined as the ability to understand complex organizational interdependencies, as both an essential skill and a critical gap, reveals a substantial limitation in medical education. Today’s healthcare leaders must navigate complex organizational interdependencies while recognizing that solutions cannot be one size fits all, yet medical education continues to prepare physicians to excel primarily as individuals rather than to think systemically about interconnected healthcare challenges [6].

Financial acumen represents perhaps the most striking disconnect between the skills required of leaders and their formal preparation. Multiple participants provided specific examples of how financial disengagement or limited understanding can reduce the impact physicians are able to have. This finding extends beyond prior research on business education motivations, revealing that financial literacy directly influences physicians’ ability to shape organizational decisions and effectively advocate for patients [7,8].

Development gaps: critical skills, delayed training

Our investigation exposes a troubling paradox: the leadership competencies most critical for healthcare effectiveness are precisely those that current leaders wish they had developed much earlier in their careers. This disconnect is perhaps best captured by one participant's insight that “80% of a physician leader’s future success depends on their ability to develop meaningful relationships with others, while only 20% depends on their technical skills as a doctor.” This insight underscores the gap between medical training priorities and actual leadership demands [1,9].

Relational leadership emerged as both the most essential skill and the most significant development gap among current leaders. Participants emphasized that while physicians excel in clinical knowledge, they often struggle with the interpersonal demands of leadership because they are not trained to lead and manage other people. This training gap becomes particularly problematic as physicians advance into roles where success depends heavily on developing meaningful relationships with others rather than on individual technical expertise.

The gap in self-awareness and emotional regulation represents another critical deficiency in leadership preparation. Leaders consistently emphasized that effective leadership begins with managing one’s own emotional responses, particularly during high-stress situations. Yet medical training assumes rather than systematically teaches these fundamental competencies. The seemingly simple ability to avoid reactive behavior under stress or anger, as one participant noted, proves essential for healthcare leaders but receives virtually no structured development in medical education.

Medical education alignment

The root of these challenges lies in a fundamental misalignment between medical education’s approach and healthcare leadership’s demands. Traditional medical education emphasizes individual clinical excellence, while healthcare leadership increasingly requires collaborative, emotionally intelligent, and systems-oriented thinking. Even for highly capable and hardworking individuals, leadership skills do not necessarily emerge automatically from their training and experience.

The recognition that, whether they want to be leaders or not, the world will perceive physicians as leaders demands that leadership preparation become central to physician professional identity rather than an optional career path pursued by a select few [9]. This shift requires medical schools to rethink and expand their educational mission beyond the development of individual clinical competency.

Our findings also reveal a promising pathway forward through experiential learning. Participants consistently emphasized that the most growth comes from real-world experience and that leadership skills transfer effectively across different contexts and levels of responsibility. The transferability of leadership skills suggests that medical education could provide authentic leadership development opportunities without requiring entirely new curriculum structures [5,10].

Implications for healthcare leadership development

We hope that our findings will inspire a new consideration of physician leader preparation. For medical education, the evidence supports integrated leader development throughout training, rather than additive programming [5,10]. Leadership must become core to professional identity formation from matriculation, not an optional career path pursued by select individuals. In addition, our work supports experiential learning approaches and the development of transferable leadership skills that enable individuals to lead at larger scales and have greater impact.

The need for financial literacy deserves particular attention, given its critical importance for maintaining physician influence in healthcare decision-making. Rather than treating business education as separate from clinical preparation, medical schools should consider financial literacy an essential component of patient advocacy and high-quality care delivery [11].

Healthcare organizations, meanwhile, must begin identifying and developing leaders much earlier in physicians’ careers rather than waiting until they assume formal leadership positions. The recognition that leader development occurs over decades, much like medical training itself, suggests the need for structured, longitudinal approaches that begin in medical school and continue throughout physicians’ careers [5]. Organizations can create opportunities for emerging leaders to practice essential competencies in supported environments while receiving guidance from experienced leaders, which may effectively bridge current development gaps [2,5,12].

Limitations and future directions

This study has several important limitations that warrant consideration in interpreting these findings. Our focus on senior leaders within North Carolina healthcare systems may not fully capture experiences across different regions or organizational contexts. The timing of data collection, which occurred during a period of significant healthcare transformation, likely influenced participants’ emphasis on adaptive capabilities, although this context also provided valuable insights into leadership requirements during system-wide change. Future research should examine the effectiveness of integrated leadership development approaches through longitudinal studies tracking career impact, investigate specific approaches to developing emotional intelligence and systems thinking in medical trainees, and conduct comparative analyses across different organizational contexts to understand how leadership needs vary by setting and specialty [1,5,6].

## Conclusions

Clinical expertise alone is not sufficient for leading modern healthcare organizations. This investigation reveals critical opportunities for enhancing physician leadership development through the systematic integration of interpersonal, adaptive, and technical competencies. As healthcare organizations continue confronting ongoing challenges and transformations, they require leaders with sophisticated capabilities that bridge clinical excellence with collaborative leadership skills. The path forward requires medical schools and healthcare organizations to fundamentally reconstruct leader development, beginning early in medical training and continuing throughout physicians’ careers. Physicians are perceived as leaders, whether they hold a title or position of leadership. The role of physicians as leaders should be part of their professional identity and training. Preparing physicians for their roles as leaders represents both an educational imperative and a critical investment in the future of healthcare.
